# Minocycline Activity against Unusual Clinically Significant Gram-Negative Pathogens

**DOI:** 10.1128/AAC.01264-21

**Published:** 2021-10-18

**Authors:** Dee Shortridge, S. J. Ryan Arends, Jennifer M. Streit, Mariana Castanheira

**Affiliations:** a JMI Laboratories, North Liberty, Iowa, USA

**Keywords:** *Achromobacter*, *Aeromonas*, minocycline, surveillance

## Abstract

The minocycline susceptibility of 3,856 isolates including *Burkholderia*, *Achromobacter*, *Alcaligenes*, *Aeromonas*, and Stenotrophomonas maltophilia from the SENTRY surveillance (2014 to 2019) were analyzed. The susceptibilities of these species (%S) were *Achromobacter* spp. (*n* = 411; 92.6%), Burkholderia cepacia species complex (*n* = 199; 85.9%), *Aeromonas* spp. (*n* = 127; 99.2%), *Chryseobacterium* spp. (*n* = 59; 94.9%), Alcaligenes faecalis (*n* = 42; 88.1%), and S. maltophilia (*n* = 2,287; 99.5%). These data suggest that minocycline is a useful treatment option for infections caused by unusual Gram-negative pathogens.

## INTRODUCTION

Unusual Gram-negative (GN) pathogens, including *Achromobacter* spp., *Alcaligenes* spp., *Aeromonas* spp., *Burkholderia* spp., and other genera, are primarily opportunistic pathogens that can cause serious infections (1–3). *Achromobacter* spp., particularly A. xylosoxidans, have been isolated from cystic fibrosis (CF) patients, and the incidence has increased recently (4–6). *Aeromonas* spp. have been associated with necrotizing fasciitis (7, 8). Burkholderia cepacia species complex and Stenotrophomonas maltophilia have been recognized as causes of bloodstream infections and pneumonia in immunocompromised patients (9, 10). Minocycline *in vitro* activity has been published in recent studies on Acinetobacter baumannii*-calcoaceticus* species complex, B. cepacia complex, and S. maltophilia (10–13). However, recent publications with susceptibilities of the less frequently isolated GN isolates are uncommon (14). In this study, we analyzed the susceptibilities of uncommon species to minocycline and comparators.

From 2014 to 2019, 3,856 unusual GN isolates were consecutively collected from hospitalized patients as part of the global SENTRY Antimicrobial Surveillance program (15). Briefly, laboratories submitted 1 isolate per patient per infection episode. Chart reviews were not performed to determine if the isolate was a colonizer rather than a pathogen. Identifications were performed by the submitting laboratory and confirmed at JMI Laboratories with matrix-assisted laser desorption ionization-time of flight mass spectrometry (MALDI-TOF; current BioTyper Compass software version 4.1.100.1; Bruker Daltonics, Billerica, MA, USA). BioTyper software updates were applied as available from the manufacturer throughout the surveillance period.

The most common GN species, Pseudomonas aeruginosa and Acinetobacter baumannii*-calcoaceticus* complex, were excluded from this analysis, as the focus was on less commonly isolated species. Each genus selected for analysis had at least 10 isolates. Some genera had multiple species, each with a small number of isolates that were combined for analysis; therefore, these results should be interpreted with caution.

Isolates were tested for susceptibility to minocycline and comparators using frozen-form broth microdilution (16). CLSI M100 or M45 breakpoints were used, as appropriate (17, 18). If minocycline breakpoints were not available for an organism, CLSI breakpoints for “other non-*Enterobacterales*” (≤4/8/≥16 mg/liter) were applied.

Pneumonia in hospitalized patients (PIHP) was the most frequent infection from which the organisms were isolated, including Stenotrophomonas maltophilia (*n* = 1,586/2,285; 69.4%), *Achromobacter* spp. (*n* = 290/411; 70.6%), non-*aeruginosa* Pseudomonas (*n* = 129/340; 38.1%), and *Burkholderia* spp. (*n* = 180/243; 73.2%). Non-*baumannii-calcoaceticus* Acinetobacter spp. were primarily isolated from bloodstream infections (*n* = 179/422; 42.4%). *Aeromonas* spp. were primarily isolated from skin and soft tissue infections (*n* = 49/127; 38.6%), closely followed by bloodstream infections (*n* = 41/127; 32.3%). Alcaligenes faecalis was primarily isolated from skin and soft tissue infections (*n* = 30/42; 71.4%), while *Chryseobacterium* spp. and *Elizabethkingia* spp. were primarily isolated from PIHP (*n* = 39/59 [59.3%] and *n* = 16/23 [69.5%], respectively). Isolates were primarily from the United States (54.3%) and Europe (36.4%).

The MIC frequency distribution and MIC_50/90_ values of minocycline for the genera analyzed are shown in Table 1. Susceptibility for all isolates was 97.0% (3,739/3,856) and was over 90% for the genera shown, except for Alcaligenes faecalis, *Burkholderia* spp., and non*-aeruginosa* Pseudomonas spp. Minocycline susceptibility of *A. faecalis* was 88.1%, *Burkholderia* spp. was 86.8%, and B. cepacia species complex was 86.3%. The susceptibility of non*-aeruginosa* Pseudomonas spp. was 89.7%.

**TABLE 1:** MIC distribution of minocycline tested against various unusual nonfermentative Gram-negative species with at least 10 isolates

Organism (no. of isolates)	Minocycline MIC (mg/liter) at^*a*^:									MIC_50_ (mg/liter)	MIC_90_ (mg/liter)
	0.06	0.12	0.25	0.5	1	2	4	8	>8		
Acinetobacter non*-baumannii* complex. (422)	166 (39.2)	129 (70.0)	80 (88.9)	32 (96.5)	9 (98.6)	0 (98.6)	**1 (98.8)**	1 (99.1)	4 (100.0)	0.12	0.5
*Achromobacter* spp. (411)	2 (0.5)	7 (2.2)	21 (7.3)	62 (22.4)	141 (56.7)	97 (80.3)	**51 (92.7)**	22 (98.1)	8 (100.0)	1	4
*Aeromonas* spp. (127)	0 (0.0)	2 (1.6)	10 (	9.4)60 (56.7)	35 (84.3)	16 (96.9)	**3 (99.2)**	0 (99.2)	1 (100.0)	0.5	2
*A. hydrophila* (35)	0 (0.0)	1 (2.9)	4 (14.3)	17 (62.9)	10 (91.4)	2 (97.1)	**1 (100.0)**			0.5	1
Alcaligenes faecalis (42)		0 (0.0)	1 (2.4)	1 (4.8)	8 (23.8)	19 (69.0)	**8 (88.1)**	3 (95.2)	2 (100.0)	2	8
*Burkholderia* spp. (243)	1 (0.4)	1 (0.8)	1 (1.2)	14 (7.0)	57 (30.5)	89 (67.1)	**48 (86.8)**	12 (91.8)	20 (100.0)	2	8
B. cepacia *complex* (226)	1 (0.40)	0 (0.40)	1 (0.90)	13 (6.6)	51 (29.2)	82 (65.5)	**47 (86.3)**	11 (91.2)	20 (100.00)	2	8
Non*-cepacia Burkholderia* spp. (17)	0 (0.0)	1 (5.9)	0 (5.9)	1 (11.8)	6 (47.1)	7 (88.2)	**1 (94.1)**	1 (100.0)		2	4
*Chryseobacterium* spp. (59)	0 (0.0)	1 (1.7)	0 (1.7)	6 (11.9)	14 (35.6)	21 (71.2)	**14 (94.9)**	3 (100.0)		2	4
*Delftia* spp. (13)	1 (7.7)	2 (23.1)	5 (61.5)	5 (100.0)						0.25	0.5
*Elizabethkingia* spp. (23)		0 (0.0)	1 (4.3)	12 (56.5)	8 (91.3)	1 (95.7)	**0 (95.7)**	1 (100.0)		0.5	1
*Ochrobactrum* spp. (16)	0 (0.0)	1 (6.2)	1 (12.5)	8 (62.5)	6 (100.0)					0.5	1
Non*-aeruginosa* Pseudomonas spp. (340)	0 (0.0)	1 (0.3)	9 (2.9)	24 (10.0)	76 (32.4)	119 (67.4)	**76 (89.7)**	20 (95.6)	15 (100.0)	2	8
*Rhizobium* spp. (15)	3 (20.0)	6 (60.0)	5 (93.3)	0 (93.3)	1 (100.0)					0.12	0.25
*Shewanella* spp. (14)	0 (0.0)	1 (7.1)	6 (50.0)	6 (92.9)	1 (100.0)					0.25	0.5
*Sphingomonas* spp. (19)	13 (68.4)	4 (89.5)	0 (89.5)	1 (94.7)	1 (100.0)					≤0.06	0.5
Stenotrophomonas maltophilia (2,285)	4 (0.2)	87 (4.0)	491 (25.5)	973 (68.1)	443 (87.4)	195 (95.9)	**81 (99.5)**	9 (99.9)	2 (100.0)	0.5	2.0
Non*-maltophilia Stenotrophomonas* spp. (12)	2 (16.7)	6 (66.7)	1 (75.0)	3 (100.0)						0.12	0.5

a CLSI (M100 or M45) minocycline susceptible breakpoint (4 mg/liter) is indicated in the column with boldfaced text.

The MIC_50/90_ values and susceptibilities to minocycline and comparators, including meropenem and meropenem-vaborbactam, for the largest organism groups are shown in Table 2. Minocycline had the highest susceptibility (98.8%) of the agents tested against Acinetobacter spp., followed by levofloxacin at 97.2%. Imipenem and meropenem also had 95% or greater susceptibility.

**TABLE 2:** Activity of minocycline and comparator antimicrobial agents tested against uncommon Gram-negative species collected from 2014–2019

Organism/antimicrobial	MIC_50_ (mg/liter)	MIC_90_ (mg/liter)	MIC range (mg/liter)	CLSI criterion^*a*^ (%)		
				S	I	R
Acinetobacter spp.^*b*^ (*n* = 422)						
Minocycline	0.12	0.5	≤0.06–>8	98.8	0.2	0.9
Meropenem-vaborbactam	0.25	1	≤0.015–>32			
Meropenem	0.25	1	≤0.015–>32	95.0	0.5	4.5
Imipenem	≤0.12	0.5	≤0.12–>8	95.3	0.2	4.5
Piperacillin-tazobactam	≤0.5	64	≤0.5–>64	88.1	3.1	8.8
Tetracycline	2	4	≤0.5–>8	94.1	2.2	3.6
Amikacin	1	4	≤0.25–>32	95.3	1.7	3.1
Ceftazidime	4	>16	≤0.25–>16	79.5	8.3	12.3
Colistin	≤0.5	2	≤0.5–>8		90.5^*c*^	9.5
Gentamicin	≤1	2	≤1–>8	94.3	1.7	4
Levofloxacin	≤0.12	0.5	≤0.12–>4	97.2	0.9	1.9
*Achromobacter* spp.^*d*^ (*n* = 411)						
Minocycline	1	4	≤0.06–>8	92.7	5.4	1.9
Meropenem-vaborbactam	0.12	4	0.03–>32			
Meropenem	0.25	16	0.03–>32	85.9	3.9	10.2
Imipenem	1	4	0.25–>8	90.3	4.9	4.9
Amikacin	>32	>32	≤0.25–>32	9.5	5.6	84.9
Aztreonam	>16	>16	2–>16	1.0	1.0	98.1
Cefepime	>16	>16	≤0.12–>16	10.7	27.3	62.0
Ceftazidime	4	16	0.25–>16	76.9	13.1	10.0
Colistin	1	4	≤0.5–>8			
Gentamicin	>8	>8	≤0.12–>8	6.1	2.7	91.2
Levofloxacin	4	>4	≤0.12–>4	36.0	30.7	33.3
Piperacillin-tazobactam	≤0.5	16	≤0.5–>64	92.2	3.6	4.1
Tetracycline	>8	>8	≤0.5–>8	16.7	3.0	80.3
Trimethoprim-sulfamethoxazole	≤0.5	4	≤0.5–>4	86.1		13.9
Burkholderia cepacia complex (*n* = 226)						
Minocycline	2	8	≤0.06–>8	86.3	4.9	8.8
Meropenem-vaborbactam	0.5	2	0.03–>32			
Meropenem	2	8	0.03–>32	88.9	5.8	5.3
Amikacin	>32	>32	≤0.25–>32			
Cefepime	16	>16	≤0.5–>16			
Ceftazidime	2	16	0.12–>16	87.1	5.8	7.1
Colistin	>8	>8	≤0.5–>8			
Gentamicin	>8	>8	≤1–>8			
Levofloxacin	2	>4	≤0.12–>4	65.5	16.4	18.1
Piperacillin-tazobactam	4	64	≤0.5–>64			
Tetracycline	>8	>8	1–>8			
Trimethoprim-sulfamethoxazole	≤0.5	4	≤0.5–>4	89.8		10.2
Pseudomonas spp.^*e*^ (*n* = 340)						
Minocycline	2	8	0.12–>8	89.7	5.9	4.4
Meropenem-vaborbactam	2	8	≤0.015–>32			
Meropenem	2	8	≤0.015–>32	85.6	9.7	4.7
Imipenem	0.5	4	≤0.12–>8	93.8	3.8	2.4
Tetracycline	4	8	≤0.5–>8	78.7	13.4	7.8
Amikacin	1	2	≤0.25–>32	97.6	0.6	1.8
Aztreonam	16	>16	≤0.12–>16	26.2	24.4	49.4
Cefepime	2	8	≤0.5–>16	92.6	3.2	4.1
Ceftazidime	2	8	≤0.25–>16	90.9	3.5	5.6
Colistin	≤0.5	2	≤0.5–>8			
Gentamicin	≤1	2	≤1–>8	94.1	1.2	4.7
Levofloxacin	0.5	4	≤0.12–>4	89.1	2.6	8.2
Piperacillin-tazobactam	8	16	≤0.5–>64	90.6	7.6	1.8
Stenotrophomonas maltophilia (*n* = 2,287)						
Minocycline	0.5	2	≤0.06–>8	99.5	0.4	0.1
Meropenem-vaborbactam	>32	>32	≤0.015–>32			
Meropenem	>32	>32	≤0.015–>32			
Ceftazidime	>16	>16	0.25–>16	24.0	8.8	67.2
Colistin	4	>8	≤0.5–>8			
Gentamicin	>8	>8	≤1–>8			
Levofloxacin	1	>4	≤0.12–>4	79.6	9.2	11.2
Tetracycline	>8	>8	2–>8			
Trimethoprim-sulfamethoxazole	≤0.5	≤0.5	≤0.5–>4	95.0		5.0

a Criteria as published by CLSI (18).

b Organisms include Acinetobacter beijerinckii (3), *A. berezinae* (34), *A. courvalinii* (6), *A. guillouiae* (8), *A. haemolyticus* (14), *A. johnsonii* (20), A. junii (27), A. lwoffii (78), A. nosocomialis (8), A. pittii (35), *A. proteolyticus* (2), *A. radioresistens* (49), *A. schindleri* (3), *A. soli* (10), *A. towneri* (2), *A. ursingii* (83), *A. variabilis* (6), *A. vivianii* (1), and unspeciated Acinetobacter (33).

c CLSI removed the susceptible criteria for colistin; all isolates with MIC values of ≤2 mg/liter are considered colistin intermediate (18).

d Organisms include Achromobacter denitrificans (12), *A. insolitus* (6), *A. marplatensis* (1), A. xylosoxidans (202), and unspeciated *Achromobacter* (190).

e Organisms include Pseudomonas alcaligenes (3), *P. alcaliphila* (2), P. chlororaphis *group* (1), *P. citronellolis* (5), P. fluorescens (14), P. fluorescens *group* (38), P. fluorescens*/putida* (5), *P. fulva* (6), *P. guariconensis* (2), *P. koreensis* (11), *P. luteola* (2), P. mendocina (19), *P. monteilii* (5), *P. mosselii* (5), *P. nitroreducens/multiresinivorans group* (3), *P. oleovorans/pseudoalcaligenes group* (1), *P. oryzihabitans* (7), P. otitidis (6), *P. peli* (2), P. plecoglossicida (6), P. protegens (3), *P. psychrotolerans* (2), P. putida (35), P. putida *group* (84), P. stutzeri (32), and unspeciated Pseudomonas (41).

Minocycline and piperacillin-tazobactam had the highest susceptibilities against *Achromobacter* species, with 92.7% and 92.2% susceptible, respectively (Table 2). Imipenem and meropenem susceptibilities were 90.3% and 85.9%, respectively, and trimethoprim-sulfamethoxazole susceptibility was 86.1%. *Achromobacter* species isolates were resistant to the aminoglycosides amikacin and gentamicin and had poor susceptibility to aztreonam (1.0%), cefepime (10.7%), and levofloxacin (36.0%).

Most (93.1%) *Burkholderia* species isolates were B. cepacia complex (Table 2). Of the agents tested, 5 antimicrobials have CLSI breakpoints for *Burkholderia* spp. (17, 18). Trimethoprim-sulfamethoxazole had the highest susceptibility of 89.8%; the susceptibilities of meropenem, ceftazidime, and minocycline were 88.9%, 87.1%, and 86.3%, respectively.

Of the 340 non*-aeruginosa* Pseudomonas species isolates, the P. putida group (*n* = 84) was the most common species (Table 2). As CLSI breakpoints for P. aeruginosa are no longer applicable to non-*aeruginosa* species, the CLSI breakpoints for other non-*Enterobacterales* were applied (18). Minocycline inhibited 89.7% of isolates with a MIC value of ≤4 mg/liter. The two drugs with the highest susceptibilities were amikacin (97.6%) and gentamicin (94.1%).

Minocycline susceptibility against S. maltophilia was 99.5%, the highest of the four antimicrobials tested with CLSI breakpoints (Table 2). Trimethoprim-sulfamethoxazole susceptibility was 95.0%, levofloxacin was 79.6%, and ceftazidime was 24.0%.

Although minocycline was first introduced in 1967, its use for the treatment of infections caused by unusual GN has increased due to its potent *in vitro* activity, good tissue penetration, and low toxicity (19–22). In addition, the approval of an improved intravenous formulation in the United States in 2015 facilitated its use in hospitalized patients with serious infections (22–24). Combination therapy with minocycline and various antimicrobials, including colistin and cefiderocol, have also been studied, although there is no consensus about which combinations are the most useful (25–27).

Overall, minocycline susceptibility was greater than 85% for the various species tested, including 99.2% susceptibility for *Aeromonas* spp., 98.8% for non*-baumannii* Acinetobacter, 92.7% for *Achromobacter* spp., and 99.5% for S. maltophilia. While colistin was also active against several species, its clinical use is discouraged due to toxicity and poor efficacy (28). Trimethoprim-sulfamethoxazole also had good *in vitro* activity against *Achromobacter* spp., *Burkholderia* spp., and S. maltophilia. Resistance to trimethoprim-sulfamethoxazole has been reported in S. maltophilia (29, 30). In this study, 5.0% of isolates were resistant to trimethoprim-sulfamethoxazole and 92.9% of those isolates were susceptible to minocycline.

This study has several limitations: the recently approved drugs cefiderocol and eravacycline may have activity against these isolates but were not tested; most of the isolates analyzed were from the United States; there was no medical chart review, so it is unknown if any isolates were colonizers rather than pathogens; MALDI-TOF was used for isolate identification, which may not distinguish among relevant species of some analyzed genera; and no molecular characterization was performed.

The number of antimicrobials that the clinical laboratory can test against unusual GN pathogens and report interpretive criteria for is limited. This study provides useful susceptibility information for several genera that are less frequently included in publications. These *in vitro* data suggest that minocycline remains a useful treatment option for infections caused by unusual GN.
